# Sodium Butyrate Alleviates Lipopolysaccharide-Induced Inflammatory Responses by Down-Regulation of NF-κB, NLRP3 Signaling Pathway, and Activating Histone Acetylation in Bovine Macrophages

**DOI:** 10.3389/fvets.2020.579674

**Published:** 2020-11-05

**Authors:** Liqiang Jiang, Jingjing Wang, Ziyi Liu, Aimin Jiang, Shuangqiu Li, Di Wu, Yong Zhang, Xingyi Zhu, Ershun Zhou, Zhengkai Wei, Zhengtao Yang

**Affiliations:** ^1^College of Life Sciences and Engineering, Foshan University, Foshan, China; ^2^College of Veterinary Medicine, Jilin University, Changchun, China

**Keywords:** sodium butyrate, inflammation, NF-κB, H3K9, bovine macrophage

## Abstract

Sodium butyrate is the sodium salt of butyric acid, which possesses many biological functions including immune system regulation, anti-oxidant and anti-inflammatory ability. The present study was designed to elucidate the anti-inflammatory effects and mechanisms of sodium butyrate on lipopolysaccharide (LPS)-stimulated bovine macrophages. The effect of sodium butyrate on the cell viability of bovine macrophages was assayed by using the CCK-8 kit. Quantitative real-time PCR (qRT-PCR) was used to detect the gene expression of interleukin-6 (IL-6), interleukin-1β (IL-1β), cyclooxygenase-2 (COX-2), and inducible Nitric Oxide Synthase (iNOS). NF-κB, NLRP3 signaling pathway, and histone deacetylase were detected by western blotting. The results showed that sodium butyrate had no significant effect on cell viability at 0–1 mM, and inhibited LPS-induced IL-6, IL-1β, COX-2, and iNOS expression. Moreover, sodium butyrate suppressed LPS (5 μg/ml)-stimulated the phosphorylation of IκB and p65, inhibited the deacetylation of histone H3K9, and has also been found to inhibit protein expression in NLRP3 inflammasomes. Thus, our finding suggested that sodium butyrate relieved LPS-induced inflammatory responses in bovine macrophage by inhibiting the canonical NF-κB, NLRP3 signaling pathway, and histone decetylation, which might be helpful to prevent cow mastitis.

## Introduction

Bovine mastitis is one of the most common diseases in dairy farming, which causes great economic losses to the world dairy industry (1). There are many factors that can cause mastitis, including environmental conditions, milking methods, and microbial infections. However, mastitis caused by pathogenic bacteria is the most common in daily productions. Among microorganisms, Gram-negative bacteria such as *Escherichia coli* can cause severe mastitis in dairy cows, seriously affecting the health and milk quality of cows (2). Lipopolysaccharide (LPS) is an active component of the cell wall of gram-negative bacteria. When these bacteria rupture, they enter the animal body and promote the development of inflammation (3). Therefore, LPS is regarded as the major factor to induce mastitis when cows are infected by gram-negative bacteria in mammary gland. Besides, LPS-induced mastitis model has been frequently used to study the prevention and treatment of this disease. During inflammation, circulating monocytes migrate from the blood to tissues where they differentiate into macrophages that are able to phagocytosis and production of both pro-inflammatory and anti-inflammatory cytokines (4). In the bovine udder, macrophages are present in the mammary gland interstitium and acinus cells protecting mammary epithelium against invading microorganisms (5). Thus, macrophages in the mammary gland are an essential immunological defense mechanism against infection in the innate immune system.

The dietary fiber of animal intestinal microbial fermentation can produce a variety of short-chain fatty acids (SCFA), which are important for the protection and nutrient absorption of animal intestines (6). SCFA have a variety of functions, such as immune regulation and anti-tumor effect, as well as anti-inflammatory effect (7–9). Some studies have verified that short-chain fatty acids inhibit histone deacetylase (HDAC) and thus regulate colitis in animals (10–12). Since Butyric acid has been demonstrated to show positive effects on control of enteric pathogens, reduction of inflammation, and modulation of gut microbiota, particular attention has been paid on sodium butyrate. Butyric acid, a kind of short-chain fatty acid, has been reported to inhibit the activation of NLRP3 inflammasome (13, 14) and to play a beneficial role in obesity and cardiovascular inflammation (15–18). During inflammation, nuclear transcription factor NF-κB induces inflammatory cells to express and secrete a variety of pro-inflammatory mediators, such as COX-2, iNOS, IL-6, etc. (10, 19) and also regulates the formation of inflammasome which is a protein complex responsible for promoting IL-1β maturation and responds to a variety of stimuli including LPS (20–24). However, few sodium butyrate has been reported in bovine macrophage inflammation. In the present study, the aim was to investigate the role of sodium butyrate in LPS-induced inflammation in bovine macrophages and to examine its underlying mechanisms.

## Materials and Methods

### Bovine Macrophage Culture

The bovine macrophage cell line used in this study was provided by Professor Guo Aizhen from Huazhong Agricultural University in Wuhan, China, and was grown as described previously (25, 26). Bovine macrophages were cultured in transparent culture 25 cm^2^ flaskscontaining RPMI 1640 medium (Hyclone, USA) supplemented with 10% fetal bovine serum (FBS, Biological Industries, Israel) and double antibiotics (1% penicillin and streptomycin, Hyclone, USA), and were incubated in an environment at a constant temperature of 37°C and 5% carbon dioxide. Culture media were changed every day. After about 72 h, cells overgrew on the contact surface of the cell culture flask, 1 mL trypsinsolution was added to detach cells from the bottle wall, and the culture media containing serum was immediately added to terminate the digestion, then subculture was performed. Generally, cells were cultured for 2–3 generations for experiments.

### Cell Viability Assay

The effect of sodium butyrate on the viability of bovine macrophages was tested by CCK-8 (2-(2-methoxyl-4-nitrophenyl)-3-(4-nitrophenyl)-5-(2, 4-disulfonyl benzene)−2h-tetrazolium monosalate) kit. Briefly, cells were collected from culture flasks and added to a 96-well culture plate, each well-containing 10^5^ cells, and the plate was placed in an incubator for 10 h. After 15 h treatment with different concentrations of sodium butyrate (0, 0.25, 0.5, 1, and 2 mM), and then stimulated with 5 μg/ml LPS for 3 h, cells were then incubated with CCK-8 for 2 h. At the end, the plate was read at 450 nm in a plate reader (Tecan, Grödig, Austria), and the viability of bovine macrophages was assayed. Five replicates were performed each condition.

### Quantitative Real-Time PCR

Bovine macrophages were pretreated with different concentrations of sodium butyrate (0, 0.25, 0.5, 1 mM) for 12 h, and then treated with 5 μg/ml LPS for 3 h. Total RNA in bovine macrophages was extracted using Trizol regent (Invitrogen, Carlsbad, CA, USA) according to the manufacturer's instructions, and reverse transcription of RNA was conducted by commercial cDNA synthesis kit (ThermoFisher Scientific, Waltham, MA, USA) to obtain cDNA. Real-time quantitative PCR was performed based on the synthesized cDNA. With the 7500 Fast Real-Time PCR System (Applied Biosystems, Carlsbad, California, USA), the PCR reactions were carried out as follows: 50°C for 2 min and 95°C for 10 min followed by 40 cycles of 95°C for 15 s and 60°C for 1 min. The gene expression levels of IL-6, IL-1β, COX-2, and iNOS were analyzed by the 2^−ΔΔ*CT*^method. These data were from at least three independent experiments. The sequences of the related primers are shown in Table 1.

**Table 1:** The sequence of these used primers (cattle).

**Gene**	**Primer**	**Sequence 5^**′**^ > 3^**′**^**	**Length (bp)**
IL-6	Sense	AGTTGTGCAATGGCAATTCTGA	223
	Antisense	CCCCAGCATCGAAGGTAGA	
IL-1β	Sense	ACCTGTGTCTTTCCCGTGG	162
	Antisense	TCATCTCGGAGCCTGTAGTG	
COX-2	Sense	GCCGTCTAAACCAAACA	117
	Antisense	GTGGGACTAACTCAAGGACAA	
iNOS	Sense	CCCCTGACCTTGTTCTCG	229
	Antisense	CTTCTGCCCACTTCCTCC	
GAPDH	Sense	TGCTGTCCCTGTATGCCTCT	224
	Antisense	TTTGATGTCACGCACGATTT	
β-Actin	Sense	TCACCAACTGGGACGACA	206
	Antisense	GCATACAGGGACAGCACA	

### Protein Preparation and Western Blot Analysis

Western blot analysis was performed as previously described (27–29). In brief, bovine macrophages were inoculated in six-well plates containing RPMI-1640 medium and incubated for 10 h at 37°C and 5% carbon dioxide, then treated with various concentrations of sodium butyrate (0, 0.25, 0.5, 1 mM) for 12 h before adding 5 μg/ml LPS. After 3 h exposure to LPS, bovine macrophages were lysed on ice cubes by adding cell protein extraction buffer for 15 min, and then all samples were collected and centrifuged at 4°C, 12.000 rpm. The protein concentration was determined by the BCA method.

For western blotting, briefly, proteins were separated by SDS-polyacrylamide gel electrophoresis, and transferred to polyvinylidene difluoride (PVDF) membranes. After sealing with skim milk for 3 h, the membranes were incubated with corresponding specific primary antibodies overnight at 4°C, rinsed three times with Tris-Buffered Saline Tween-20 (TBST), and then incubated with the corresponding secondary antibody for 2 h. The source of the commercial antibody used in the experiment were H3K9 (1: 1000; #4658S, Lot: 11), IκB (1: 400; #4814S, Lot: 14), p-IκB (1: 1000; #2859S, Lot: 17), p65 (1: 600; #8242S, Lot: 1), p-p65 (1: 1000; #3033S, Lot: 8), NLRP3 (1: 1000; #15101S, Lot: 11), (Cell Signaling Technology, Danvers, MA, USA), GAPDH (1: 400; BM3876, Lot: BST173876), (Boster Biological Technology Co., USA), the results came from three independent experiments. Images were captured using a camera-based imager by applying the chemiluminescent substrate to the membranes. Then, the obtained bands were analyzed with 8-bit gray scale using Image J software (National Institutes of Health, Bethesda, MD, USA). Specifically, quantitative protein bands were selected by the square box of Image J and converted into 8-bit grayscale images. Then, the intensity of each protein band was transformed into a mountain-like image through the analysis function of the software. The area enclosed by each peak was calculated by the magic wand tool to represent the grayscale value of corresponding bands.

### Statistical Analysis

All results were expressed as mean ± standard deviation from three or more independent experiments. Western blotting band density was analyzed using Image J software. The data between groups was analyzed with ANOVA followed by Dunnett's test. *P* < 0.05 or *p* < 0.01 represents statistical significance.

## Results

### Sodium Butyrate Had No Toxic Effects on Macrophage Viability

**Figure 1:**
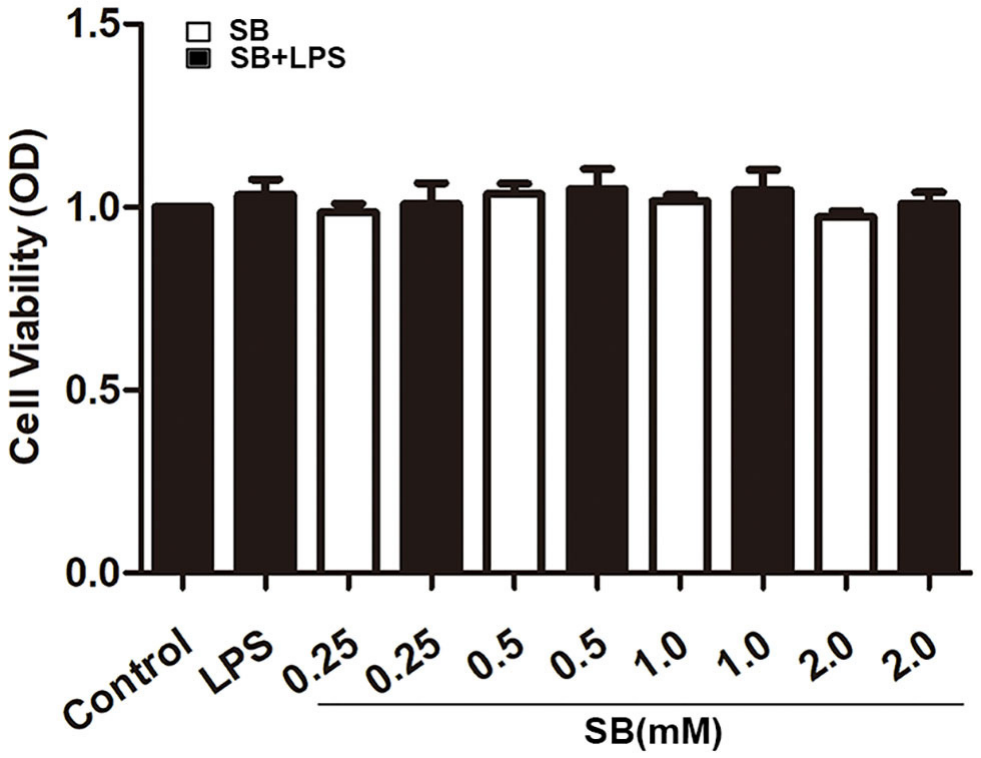
Effect of sodium butyrate on the viability of bovine macrophages. Bovine macrophages incubated with different concentrations of sodium butyrate (0.25, 0.5, 1, 2 mM) for 15 h, then stimulated with 5 μg/ml LPS for 3 h. The cell viability was assayed by CCK-8 method. Values were shown as means ±*SD* (*n* = 5).

Firstly, we checked whether sodium butyrate has influence on the viability of bovine macrophages. Confluent cells in 96-well plate were treated with different concentrations of sodium butyrate (0, 0.25, 0.5, 1, 2 mM) for 18 h, and cell viability was assayed by CCK-8 method. As shown in Figure 1, sodium butyrate as well as LPS + sodium butyrate had no significant influence on bovine macrophage viability when compared to that of untreated group, indicating its non-toxic effects on bovine macrophages.

### Sodium Butyrate Reduced Gene Expression of Inflammatory Cytokines

**Figure 2:**
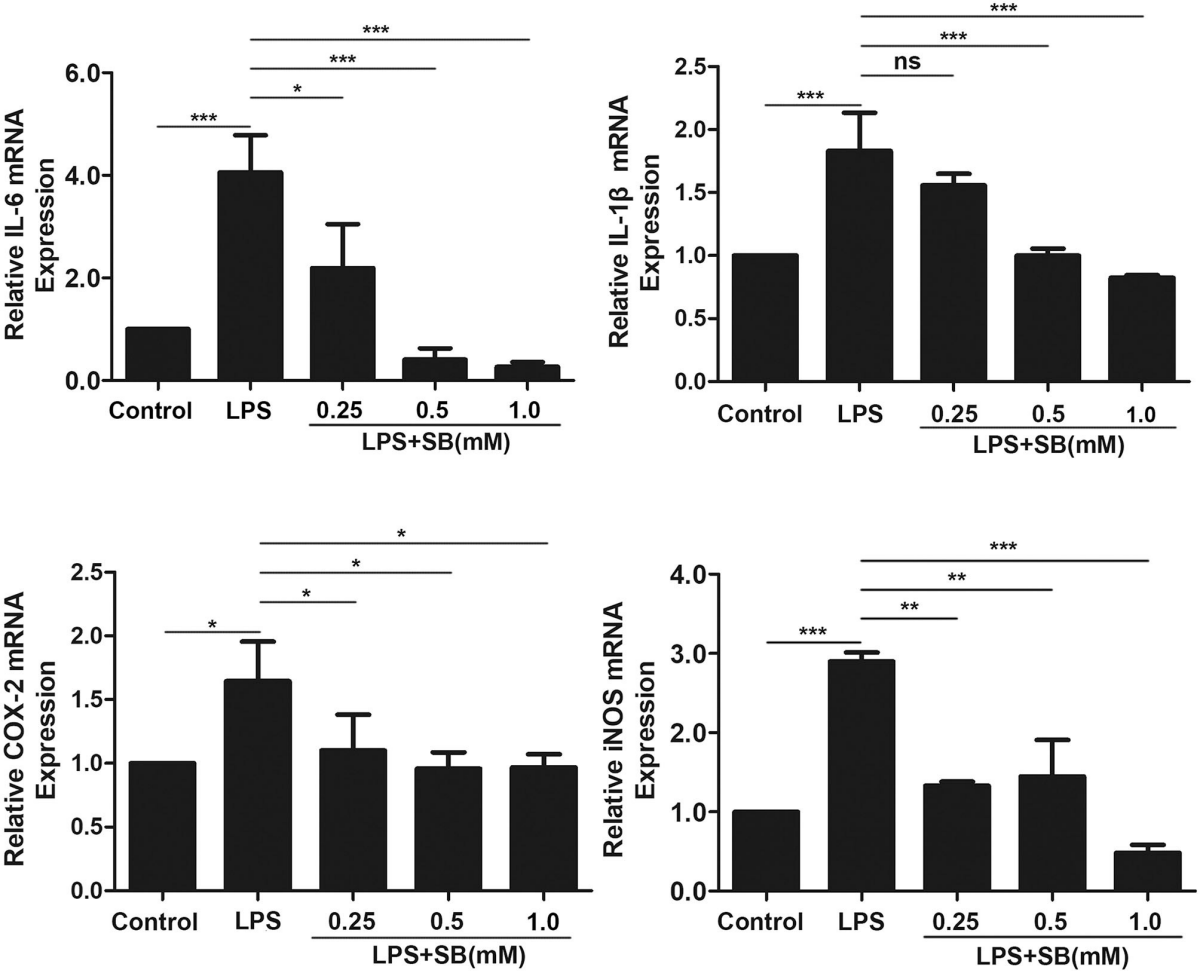
Sodium butyrate reduced gene expression of inflammatory cytokines. Bovine macrophages were pretreated with different concentrations of sodium butyrate (0.25, 0.5, 1 mM) for 12 h, and then treated with 5 μg/ml LPS for 3 h. The gene expression of IL-1β, IL-6, COX-2, and iNOS were analyzed by qRT-PCR. The gene expression levels of IL-1β, IL-6, COX-2, and iNOS are normalized to β-actin. Values were shown as means ± *SD* (*n* = 3). ^ns^*p* > 0.05 meant no significance, *p < 0.05, **p < 0.01, ***p < 0.001 compared to the LPS group.

Pro-inflammatory cytokines, such as IL-1β, and IL-6, play a key role in inflammatory diseases including bovine mastitis, and IL-1β and IL-6 are well-studied to be involved in up-regulation of inflammatory responses (30, 31). Gene expression levels of these two cytokines were obviously activated by LPS stimulation in bovine macrophages (*p* < 0.001) compared to that of control group. Nevertheless, pretreatment with sodium butyrate dose-dependently reduced this activation, especially with 0.5 and 1 mM sodium butyrate which remarkably decreased gene expression levels of IL-1β and IL-6 (*p* < 0.001) (Figure 2). COX-2 and iNOS are two crucial inducible enzymes, and their products (Prostaglandin E2 and NO) are inflammatory mediators responsible for boosting inflammatory responses in the early stages of inflammation (32). Similarly, compared to that of LPS group, mRNA expression of these two enzymes were significantly reduced by sodium butyrate pretreatment (Figure 2), indicating that sodium butyrate possibly suppresses the development of inflammation in bovine macrophages.

### Sodium Butyrate Reversed the Expression of NLRP3 Inflammasome

**Figure 3:**
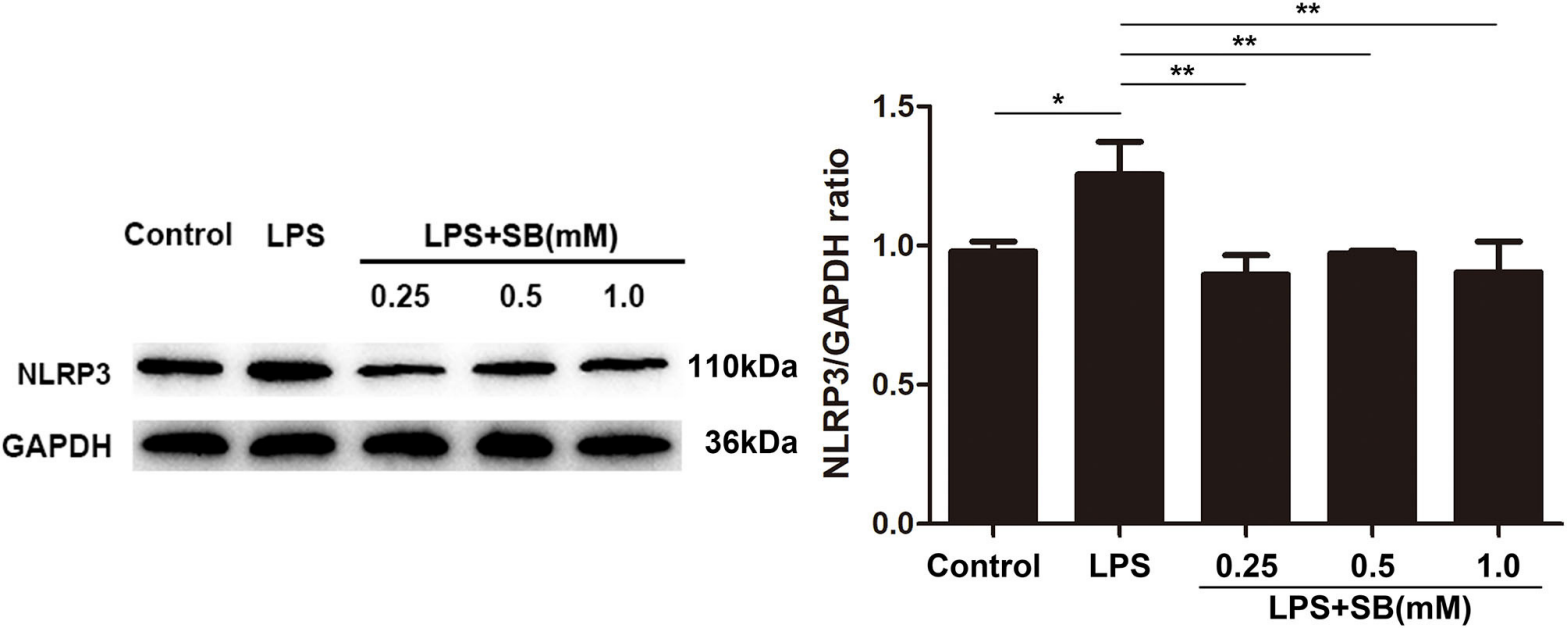
Sodium butyrate reversed the expression of NLRP3 inflammasome. After the cells were cultured with various concentrations of sodium butyrate (0.25, 0.5, 1 mM) for 12 h, and then treated with 5 μg/ml LPS for 3 h. Protein samples of NLRP3 were analyzed by Western blotting, and the quantification of the band intensity was determined by densitometry was normalized to GAPDH. Values were shown as means ± *SD* (*n* = 3). *p < 0.05, **p < 0.01 compared to the LPS group.

Inflammasomes are cytosolic multiprotein complexes responsible for pro-IL-1β and pro-IL-18 maturation (33), and NLRP3 is one of NLR proteins being involved in initiating inflammasome formation (34, 35). To further examine the anti-inflammatory mechanism of sodium butyrate, we investigated NLRP3 protein expression by western blotting. The results revealed that sodium butyrate diminished LPS-induced NLRP3 expression in a dose-dependent manner (Figure 3).

### Sodium Butyrate Inhibited NF-κB Signaling Pathway

**Figure 4:**
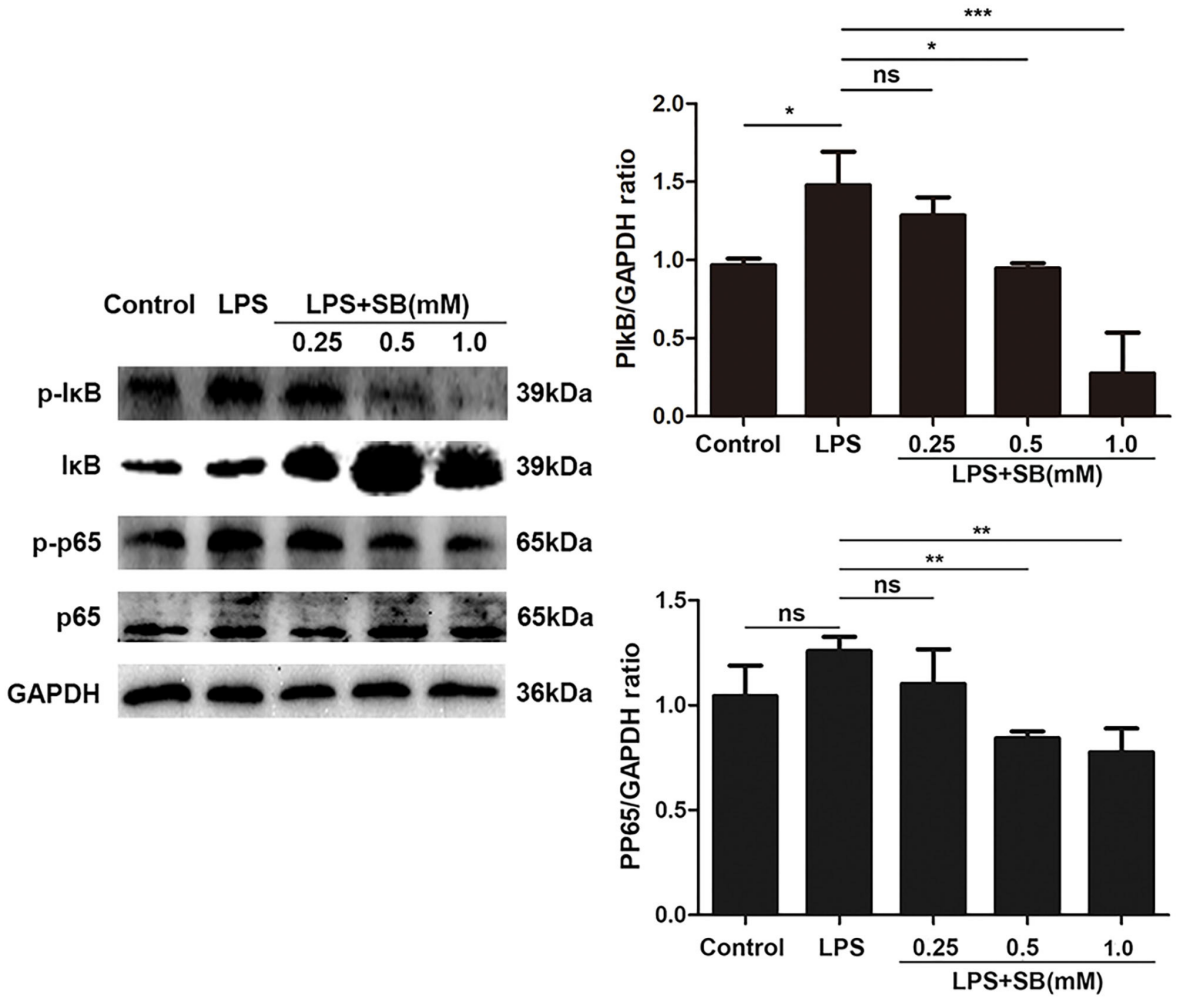
Sodium butyrate inhibited canonical NF-κB signaling pathway. Bovine macrophages were treated with different concentrations of sodium butyrate (0.25, 0.5, 1 mM) for 12 h, followed by stimulation with LPS for 3 h. Western blotting was performed to determine phosphorylation levels of IκB and p65. The quantification of band intensity was determined by densitometry and normalized to GAPDH. The values were shown as means ±*SD* (*n* = 3). ^ns^*p* > 0.05 meant no significance, *p < 0.05, **p < 0.01, ***p < 0.001 compared to the LPS group.

NF-κB is a well-known regulator of pro-inflammatory gene expression including IL-1β, IL-6, and also mediates COX-2 and iNOS expression (36). Therefore, here we evaluated the effect of sodium butyrate on nuclear transcription factor-κB. Briefly, the phosphorylation levels of NF-κB-related protein IκB and p65 were measured by Western blotting. As a result, sodium butyrate decreased phosphorylation of IκB and p65 induced by LPS in bovine macrophages, and a significant reduction was observed with 1 mM sodium butyrate (*p* < 0.05), indicating the inhibition of canonical NF-κB signaling pathway (Figure 4).

### Sodium Butyrate Increased H3K9 Acetylation

**Figure 5:**
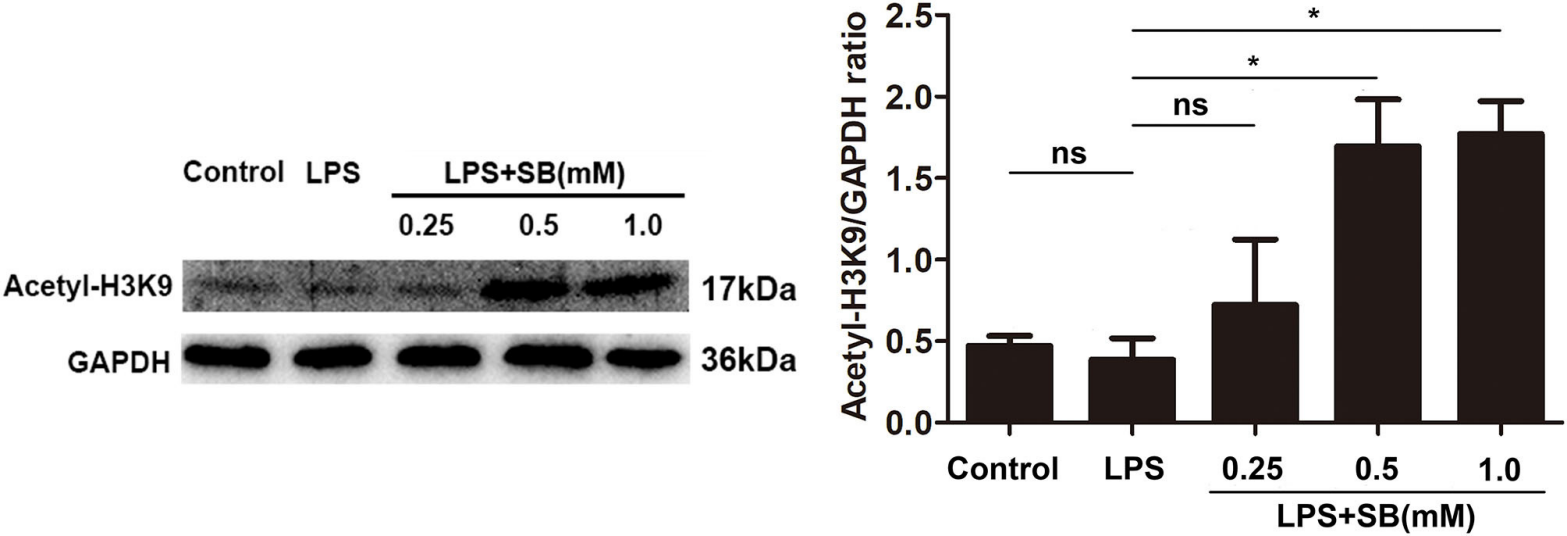
Sodium butyrate enhanced H3K9 acetylation. The cells were incubated with various concentrations of sodium butyrate (0.25, 0.5, 1 mM) for 12 h before adding LPS for 3 h. The protein level of H3K9 was analyzed by western blotting. The quantification of band intensity was determined by densitometry and normalized to GAPDH. Values were shown as means ± *SD* (*n* = 3). ^ns^*p* > 0.05 meant no significance, *p < 0.05 compared to the LPS group.

Histone acetylation is a dynamic epigenetic modification that plays important role in the regulation of gene transcription (37). It has been reported that H3K9 acetylation is beneficial to the transcription of anti-inflammatory cytokines (38). In the present study, our results showed that sodium butyrate dose-dependently increased acetylated H3K9 protein expression in LPS-induced bovine macrophages (Figure 5). Notably, sodium butyrate at 1 mM concentration dramatically elevated the level of acetylated H3K9 (*p* < 0.05).

## Discussion

Mastitis leads to a reduction in milk production in dairy cows, or even death of cows when the disease is serious, causing huge losses to the dairy industry (1). There are many factors that cause mastitis in cows, and pathogenic microbial infestation is the most important factor (2, 39). Macrophages are important immune cells in the body. They have an antagonistic effect on pathogenic microorganisms that invade the body (40). When the pathogenic microorganisms are not cleared in time, macrophages will release a variety of cytokines to aggravate the inflammatory response (2, 40, 41). Under normal physiological conditions, inflammation is an important defense response of the body against infection. The right amount of inflammatory factors and inflammatory exudate components can prevent the spread of pathogenic microorganisms, dilute toxins, and remove harmful substances (42, 43). Moreover, cells in the inflammatory area proliferate under the action of corresponding growth factors, which is beneficial to repair damaged tissues and restore the functions of tissues and organs (44). However, long-term and excessive inflammation is harmful to the body, and excessive inflammatory responses including a large amount of inflammatory exudate can damage tissues and organs and affect their functions (45). The long-term inflammation can also have a serious impact on the body, such as tumors (46, 47). Therefore, in the treatment of inflammatory diseases, in addition to eliminating the pathogenic factors, some effective measures are also taken to control the inflammatory responses. Butyrate is a short-chain fatty acid mainly produced by intestinal microbes fermenting dietary fiber. It has been reported to have immunomodulatory, anti-inflammatory and inhibit histone deacetylase functions (48, 49). Recent studies have shown that animals can change the abundance of intestinal flora after supplementing with butyrate, thereby regulating the intestinal barrier function and improving enteritis and endotoxemia (50–52), which suggested that these effects of diet are related to butyrate. In this study, by treating LPS-stimulated bovine macrophages with sodium butyrate, we found that sodium butyrate reduced the release of inflammatory factors (IL-1β, IL-6, COX-2, and iNOS) from bovine macrophages, and also inhibited the expression of inflammasome NLRP3. Furthermore, sodium butyrate significantly inhibited the phosphorylation of IκB and p65, and promoted the level of histone H3K9 acetylation in bovine macrophages.

Intramammary LPS infusion is a well-known method to establish *in vivo* model for bovine mastitis through which mastitis has been studied in a broad range of contexts (53–56). Macrophages in the mammary gland are activated by LPS infusion serving as the first defense line. Pro-inflammatory cytokines including IL-1β and IL-6 are mainly produced by activated macrophages, and play a pivotal role in the development of inflammation. Also, inflammatory mediators COX-2 and iNOS are highly expressed in LPS-stimulated macrophages. Our results revealed that sodium butyrate decreased gene expression of IL-1β and IL-6, COX-2 and iNOS demonstrating its potent anti-inflammatory effect on LPS-stimulated bovine macrophages.

Inflammasomes are an important component of the inflammatory response, while NLRP3 is a particular specific protein of inflammasomes and can be activated by a variety of factors. It has been reported that LPS can activate NLRP3 in mouse macrophages and potentiate the maturation and secretion of pro-inflammatory cytokines (such as IL-1β) (22, 23). Here we found in our experiments that sodium butyrate suppressed NLRP3 protein expression increased by LPS in bovine macrophages, further proving its strong anti-inflammatory properties.

Up-regulation of pro-inflammatory cytokines as well as iNOS and COX-2 during inflammation is mediated by the nuclear transcription factor NF-κB (57, 58). To further examine the potential anti-inflammatory mechanisms of sodium butyrate, we then investigated the effect of sodium butyrate on protein expression of NF-κB subunit p65. There are variety of TLRs receptors on the cell membrane that mediate activation of the NF-κB signaling pathway (59). When the receptor responds to ROS, LPS, etc. it activates NF-κB signaling pathway leading to release of inflammatory cytokines (60). The NF-κB dimer binds to the IκB protein. Upon activation, IκB is phosphorylated, detached from the dimer and eventually degraded, and the p65 subunit of NF-κB is transferred to the nucleus along with the dimer (61). In this present study, classical NF-κB signaling pathway in bovine macrophages was activated by LPS stimulation as indicated by elevated protein expression of phosphorylated p65 and IκB. However, sodium butyrate treatment inhibited the degradation of IκB and the activation of p65 detected by western blotting, suggesting that sodium butyrate exerts anti-inflammatory action possibly via suppressing the activation of classical NF-κB signaling pathway.

Histone modification is an epigenetic modification of non-gene sequence including methylation, acetylation, and phosphorylation (62). Histone octamer and DNA constitute nucleosomes, which are free in the N-terminal of the nucleosome and can accept various modifications, including acetylation and deacetylation (63, 64). Active histones H4 and H3 acetylate lysine residues under the action of histone acetyltransferase, which facilitates chromatin expression, while histone deacetylase acts contrary and blocks chromatin expression (65). Acetylation and deacetylation of histones are dynamic. Histone acetyltransferase promotes hyperacetylation and opens chromatin structure, allowing transcriptional activity. Histone deacetylase suppresses transcriptional activity by condensing chromatin, leading to epigenetic modification mediated expression silencing (66–68). Increasing studies have shown that short-chain fatty acids can inhibit histone deacetylase, and respond to certain gene promoter sites such as Sp1, leading to histone hyperacetylation (67). It has been shown that sodium butyrate, as (50) a histone deacetylase inhibitor, can inhibit the action of histone deacetylase, reduce the tightness of histone and DNA, and remove gene silencing, allowing transcriptional activity (10, 60, 63, 69, 70). Our previous studies have proved that propionate can act as a histone deacetylase inhibitor and promote histone H3 acetylation in mouse mammary epithelial cells (71). Here we found that sodium butyrate reversed the acetylation of H3K9 in bovine macrophages. Recently, it has been reported that histone acetylation contributes to neutrophil extracellular trap formation that is mainly composed of DNA, histone and various antimicrobial proteins (72, 73). Macrophages are also capable of releasing extracellular traps by a variety of stimuli. Thereby, we speculate that sodium butyrate possibly could exert its antimicrobial activity through inducing extracellular traps in bovine macrophages.

In summary, sodium butyrate reduced the expression of NLRP3 inflammasome, enhanced the acetylation of histone H3K9 and blocked the NF-κB signaling pathway to attenuate LPS-induced inflammatory responses in bovine macrophages, which may be helpful in the prevention and treatment of dairy cow mastitis.

## Data Availability Statement

All datasets generated for this study are included in the article/supplementary material.

## Author Contributions

LJ: data curation and writing—original draft preparation. JW: conceptualization, methodology, and software. ZL, DW, and YZ: methodology, software, and supervision. AJ and SL: methodology and supervision. XZ: supervision. EZ: funding acquisition, methodology, and software. ZW: conceptualization, methodology, and funding acquisition. ZY: project administration and funding acquisition. All authors contributed to the article and approved the submitted version.

## Conflict of Interest

The authors declare that the research was conducted in the absence of any commercial or financial relationships that could be construed as a potential conflict of interest.
